# **[**^18^F]FEPPA PET imaging for monitoring CD68-positive microglia/macrophage neuroinflammation in nonhuman primates

**DOI:** 10.1186/s13550-020-00683-5

**Published:** 2020-08-06

**Authors:** Matthew Zammit, Yunlong Tao, Miles E. Olsen, Jeanette Metzger, Scott C. Vermilyea, Kathryn Bjornson, Maxim Slesarev, Walter F. Block, Kerri Fuchs, Sean Phillips, Viktorya Bondarenko, Su-Chun Zhang, Marina E. Emborg, Bradley T. Christian

**Affiliations:** 1 Department of Medical Physics, University of Wisconsin-Madison, Madison, WI USA; 2 Waisman Center, University of Wisconsin-Madison, Madison, WI USA; 3 Preclinical Parkinson’s Research Program, Wisconsin National Primate Research Center, University of Wisconsin-Madison, 1220 Capitol Court, Madison, WI 53715 USA; 4 Cellular and Molecular Pathology Training Program, University of Wisconsin-Madison, Madison, WI USA; 5 Neuroscience Training Program, University of Wisconsin-Madison, Madison, WI USA; 6 Department of Neuroscience, University of Minnesota-Twin Cities, Minneapolis, MN USA; 7 Department of Neuroscience, University of Wisconsin-Madison, Madison, WI USA

**Keywords:** FEPPA, TSPO, CD68, Microglia, Macrophage, Neuroinflammation, Monkeys, Graft

## Abstract

**Purpose:**

The aim of this study was to examine whether the translocator protein 18-kDa (TSPO) PET ligand [^18^F]FEPPA has the sensitivity for detecting changes in CD68-positive microglial/macrophage activation in hemiparkinsonian rhesus macaques treated with allogeneic grafts of induced pluripotent stem cell-derived midbrain dopaminergic neurons (iPSC-mDA).

**Methods:**

In vivo positron emission tomography (PET) imaging with [^18^F]FEPPA was used in conjunction with *postmortem* CD68 immunostaining to evaluate neuroinflammation in the brains of hemiparkinsonian rhesus macaques (*n* = 6) that received allogeneic iPSC-mDA grafts in the putamen ipsilateral to MPTP administration.

**Results:**

Based on assessment of radiotracer uptake and confirmed by visual inspection of the imaging data, nonhuman primates with allogeneic grafts showed increased [^18^F]FEPPA binding at the graft sites relative to the contralateral putamen. From PET asymmetry analysis of the images, the mean asymmetry index of the monkeys was AI = − 0.085 ± 0.018. Evaluation and scoring of CD68 immunoreactivity by an investigator blind to the treatment identified significantly more neuroinflammation in the grafted areas of the putamen compared to the contralateral putamen (*p* = 0.0004). [^18^F]FEPPA PET AI showed a positive correlation with CD68 immunoreactivity AI ratings in the monkeys (Spearman’s *ρ* = 0.94; *p* = 0.005).

**Conclusion:**

These findings reveal that [^18^F]FEPPA PET is an effective marker for detecting increased CD68-positive microglial/macrophage activation and demonstrates sufficient sensitivity to detect changes in neuroinflammation in vivo following allogeneic cell engraftment.

## Introduction

Stem cell therapy shows great promise as a therapeutic strategy for many degenerative diseases such as Parkinson’s disease (PD) [1, 2]. Specifically, use of induced pluripotent stem cell-derived dopaminergic neurons (iPSC-mDA) as opposed to embryonic stem cells is envisioned as a sustainable, less controversial method to obtain cell lines [3, 4]. Use of same-species donor (allogeneic) iPSC lines presents the advantage of being readily available. However, the cells can trigger an immune response in the host following engraftment as a result of expression of major histocompatibility complex (MHC) molecules [5, 6]. In vivo imaging with positron emission tomography (PET) targeting activated microglia offers a promising method for monitoring the severity of the immune response following stem cell therapy, which is a critical step towards the clinical translation.

The mitochondrial translocator protein 18-kDa (TSPO) is highly expressed in activated macrophages and microglia in the brain and has been characterized as a biomarker for neuroinflammation [7–9]. As a result, there has been a strong interest in the development of PET radiotracers targeting TSPO binding to track in vivo changes in inflammatory response without requiring invasive surgery or tissue biopsy [10]. However, there are some limitations in the interpretation of microglial/macrophage activation via TSPO PET imaging [11]. Specifically, microglial activation, like macrophage activation, is classically described as pro-inflammatory (M1) or anti-inflammatory (M2) [12]. However, this concept of static M1 or M2 activation has been challenged, as the inflammatory state of microglia cells can be fluid, rapidly changing in response to the microenvironment [13]. As such, the upregulation of TSPO can be associated with both the elevated M1 and M2 states, and TSPO PET cannot distinguish between them [14], possibly because transition between states is a continuum that includes changes in cell size and morphology [13, 15, 16]. Preclinical imaging studies designed to include a direct measure of microglia/macrophages changes by *postmortem* histological analysis can provide a basis to validate whether increases in TSPO PET signal correspond to increased microglial activation. In that regard, CD68 is a well-known marker for macrophage lineage cells. CD68 expression may be present in resting microglia [17], yet it is considered a marker of activated phagocytic microglia due to its lysosome labeling. Thus, CD68 immunoreactivity is used to identify activated microglia and macrophages in the brain parenchyma and cerebral blood vessels [17].

The prototypical TSPO PET radioligands, [^11^C]-PK11195 and its enantiomer [^11^C]-(R)-PK11195, have been used to provide a measure of neuroinflammation, but their sensitivity is strongly diminished due to low brain penetration, high nonspecific binding, and high plasma protein binding [18, 19]. Thus, a variety of second-generation TSPO radioligands have been developed to improve upon the deficiencies of [^11^C]-PK11195 and provide more favorable in vivo imaging characteristics [20, 21]. Notable radioligands include [^11^C]PBR28, [^11^C]DPA713, [^11^C]ER176, [^18^F]PBR111, [^18^F]DPA714, and [^18^F]FEPPA [22, 23], all providing higher TSPO-specific binding signal, but also revealing varied sensitivity to the binding affinity state determined by the single nucleotide polymorphism rs6971 [24, 25]. [^18^F]FEPPA was developed in pursuit of a longer-lived labeling radionuclide (^18^F: t_1/2_ = 109.8 min, ^11^C: t_1/2_ = 20.3 min), providing improved counting statistics and the possibility of offsite radiopharmaceutical production and distribution [19]. FEPPA (*K*_*i*_≈0.07 nmol/L) in particular shows 3-fold higher affinity to TSPO than PBR28 (*K*_*i*_≈0.22 nmol/L) and over ten times higher affinity than DPA713 and PK11195 (*K*_*i*_≈0.87 and 1.29 nmol/L, respectively) [19]. However, the advantages of this higher TSPO affinity are partly offset due to its larger non-displaceable distribution volume [26] and yield target to off-target signal that is comparable to [^11^C]PBR28 [19, 27].

The aim of this study was to assess whether [^18^F]FEPPA PET can be used as an in vivo measure of CD68-positive microglial/macrophage activation in the brain, using a monkey model with stem cell-derived neuronal grafts. Taking advantage of an ongoing project in which hemiparkinsonian rhesus macaques were treated with allogeneic stem cell therapy, we compared in vivo [^18^F]FEPPA PET uptake to *postmortem* CD68 brain expression to validate imaging findings.

## Materials and methods

### Animals

Rhesus monkeys (*Macaca mulatta*) from the Wisconsin National Primate Research Center (WNPRC) at the University of Wisconsin-Madison, an AAALAC accredited facility, were used in this experiment. All procedures were performed in strict accordance with the recommendations in the National Research Council Guide for the Care and Use of Laboratory animals (2011) and were approved by the Institutional Animal Care and Use Committee (IACUC) at the University of Wisconsin-Madison (experimental protocol G00673). All efforts were made to minimize the number of animals used and to ameliorate any distress caused by the experimental procedures outlined in this report.

Six adult male rhesus macaques (9–13 years old, 8–19 kg) were rendered hemiparkinsonian by the administration of a unilateral (right) intracarotid artery injection of the neurotoxin 1-methyl-4-phenyl-1,2,3,6-tetrahydropyridine (MPTP) under sterile surgical conditions and isoflurane anesthesia, as previously described [28] (see Table 1 for animal information). Three to 12 months later, the monkeys received injections of allogeneic iPSC-mDA into the putamen ipsilateral to the MPTP dosing (right) under sterile surgical conditions and isoflurane anesthesia using validated methods [29]. Briefly, iPSC-mDA were generated from rhesus fibroblasts [30], genomic edited to express mCherry and intracerebrally delivered using real-time intraoperative magnetic resonance imaging (RT-IMRI) for targeting [29, 31, 32]. Each animal received approximately 15–20 million iPSC-mDA, distributed across three needle tracts with 2–3 deposits of 20–30 μL per deposit. Twenty-four months following brain surgery, the monkeys underwent [^18^F]FEPPA PET scans (details below) under isoflurane anesthesia, as previously described [33]. Approximately 72 h after [^18^F]FEPPA PET, animals were anesthetized with sodium pentobarbital (25 mg/kg iv) and transcardially perfused with heparinized saline, followed by 4% paraformaldehyde (PFA) as previously described [28, 34] to minimize variation in the response to the allograft.

**Table 1:** Information regarding rhesus macaques (*Macaca mulatta*) used in the study

Animal number	Sex	Age at necropsy (year)	Weight at necropsy (kg)	Time elapsed between MPTP dosing and grafting (month)	Time elapsed between grafting and PET (month)
1	M	9.6	14.49	12	24
2	M	10.3	14.64	3	24
3	M	11.4	15.07	12	24
4	M	11.4	11.4	3	24
5	M	9.6	15.16	12	24
6	M	13.6	15.25	3	24

*M* male, *kg* kilograms

### [^18^F]FEPPA radiochemistry

The synthesis of [^18^F]FEPPA was modified from previous methods [35, 36] to reduce precursor mass and improve high performance liquid chromatography (HPLC) separation of product from impurities. Radiolabeling was performed on an automated chemistry process control unit (CPCU). Aqueous [^18^F]-fluoride was produced with a 16-MeV GE PETtrace cyclotron in a silver target via the ^18^O(p,n)^18^F reaction using enriched [^18^O]H_2_O. The solution was passed through an Accel Plus QMA Light Sep-Pak and eluted into a reaction vessel with 700 μL of a 20% water in acetonitrile solution of potassium carbonate and kryptofix222, and rinsed with 700 μL acetonitrile. Anhydrous acetonitrile was added to the solution and dried by azeotropic distillation. FEPPA tosylate precursor (2.0 mg) was dissolved in 0.4 mL anhydrous acetonitrile and added to the dry [^18^F]-fluoride vial. Nucleophilic substitution was performed by heating at 90 °C for 10 min to produce [^18^F]FEPPA. The reaction mixture was taken up in 1.0 mL ethanol and passed through an Alumina N Plus Light Sep-Pak to remove free [^18^F]-fluoride and kryptofix222. The solution containing [^18^F]FEPPA was diluted with 0.5 mL DI H_2_O and injected onto semipreparative HPLC for purification (column: Phenomenex Luna C18, 10 μm, 250 × 10 mm; mobile phase: 20/80 acetonitrile/H_2_O + 0.5% formic acid; flow rate: 10 mL/min; UV wavelength: 254 nm). The collected fraction was diluted with 50 mL sterile water for injection, passed through a C18 Light Plus Sep-Pak, and eluted with 1.0 mL ethanol and 9.0 mL bacteriostatic saline through a 0.22-μm filter into a sterile, pyrogen-free vial. Thirty microliters of the final product was injected onto analytic HPLC (column: Intersil ODS-4, 5 μm, 150 × 4.6 mm; mobile phase: 40/60 acetonitrile/0.1 N ammonium formate; flow rate: 2.5 mL/min; UV wavelength: 254 nm) to assess the impurity profile of the final product.

### PET imaging

Twenty-four months following brain surgery, all animals underwent [^18^F]FEPPA scans in a microPET Focus 220 scanner under isoflurane anesthesia, (1–3% in 100% O_2_, 1 L/min); vital signs (respiration, temperature, heart rate) were monitored throughout as described elsewhere [33]. The monkeys were placed in the prone position with their head secured in a stereotaxic frame. After a 15-min transmission scan, radioligand was injected as an i.v. bolus (~ 185 MBq) over 30 s. Dynamic PET data were obtained for 2 h with conventionally increasing frame durations (6 × 30 s, 3 × 60 s, 2 × 120 s, 22 × 300 s). PET frames were reconstructed by 2D filtered back-projection using a ramp filter. PET images were processed and analyzed using the Statistical Parametric Mapping 12 software (SPM12; Wellcome Department of Cognitive Neurology, London, UK). Time-activity curves were generated from the reconstructed PET time series data. Image frames from the 90–120 min duration were averaged, smoothed with a 4-mm Gaussian kernel, and registered to the MRI using a resting-state frame from the RT-IMRI following the final cell deposition. The 90–120-min frame data was chosen at a sufficiently late time to avoid the bias of regional differences in radiotracer delivery (i.e., blood flow dependence), although the sensitivity to using earlier time windows was not examined. Region of interest (ROI) masks were generated for each iPSC-mDA graft site through segmentation of static frames of the RT-IMRI data collected during iPSC-mDA delivery. Precise ROIs of the grafts were drawn utilizing the signal voids in the RT-IMRI immediately following injection of the cell delivery vehicle using MRIcron (University of South Carolina, SC, USA) and were utilized for visual confirmation of [^18^F]FEPPA binding at the graft location. Masks for the ipsi/contralateral cerebellum and ipsi/contralateral putamen were generated from a rhesus atlas in standardized space [37, 38]. The RT-IMRI data was spatially normalized to the rhesus template, and the resulting deformation fields were used to transform the ROI masks of the cerebellum and putamen into the native RT-IMRI space. PET voxel activity concentrations were normalized to the injected dose and NHP weight to generate standard uptake value (SUV) images utilizing the SPM12 software. The ROI masks were applied to the PET images to extract mean SUVs for each region. An asymmetry index (Eq. 1) was calculated for the ipsilateral (SUV_R_) and the contralateral putamen (SUV_L_) to assess asymmetry in [^18^F]FEPPA between hemispheres. Using the cerebellum as a control region, an asymmetry index was calculated for the ipsi- and contralateral cerebellum to assess whether any asymmetry was a global artifact of the image.

1 $$ \mathrm{AI}=\frac{{\mathrm{SUV}}_{\mathrm{L}}-{\mathrm{SUV}}_{\mathrm{R}}}{\left({\mathrm{SUV}}_{\mathrm{L}}+{\mathrm{SUV}}_{\mathrm{R}}\right)/2} $$

### Postmortem brain tissue processing and analysis

After the brains were retrieved, they were post-fixated for 24–48 h in 4% PFA, cryoprotected in graded sucrose solutions, and then cut frozen (40 μm sections) on a standard sliding knife microtome (American Optical Corp. Model 860, Buffalo, NY, USA) as previously described [28, 34]. Serial coronal brain sections spanning the putamen from all monkeys were immunostained with antibodies against mCherry (1:2000, Thermo Fisher Scientific, Waltham, MA, USA; 1:200, secondary Goat Anti-Rat IgG Antibody, Vector Lab, Burligham, CA, USA) or CD68 (1:3000, DakoCytomation, Glostrup, Denmark; 1:200 secondary Horse Anti-Mouse IgG Antibody, Vector Lab, Burlingame, CA, USA) and counterstained with Nissl to visualize brain anatomy. Immunostaining of tissue sections from all animals was performed in parallel and included negative and positive controls for each antibody [28, 34].

Coronal brain sections immunostained against mCherry and CD68 were evaluated in a Zeiss Axioplan 2 imaging photomicroscope coupled to a MAC5000 high precision computer-controlled x-y-z motorized stage, and a MicroFire CX9000 camera. To minimize potential bias, an independent investigator evaluated mCherry immunostained tissue and selected the commissural and post-commissural putamen for evaluation of microglia/macrophage cell response. A different investigator, blind to the treatment, assessed the ipsi- and contralateral putamen of all subjects in three representative coronal brain sections (starting at the level of the anterior commissure and 960 μm apart) per subject that corresponded to the core of the graft. Tissue sections were first evaluated at low magnification (× 10) to identify areas of CD68 immunoreactivity (CD68-ir). High magnification (× 40) was then used to assess microglia/macrophage morphology.

**Fig. 1:**
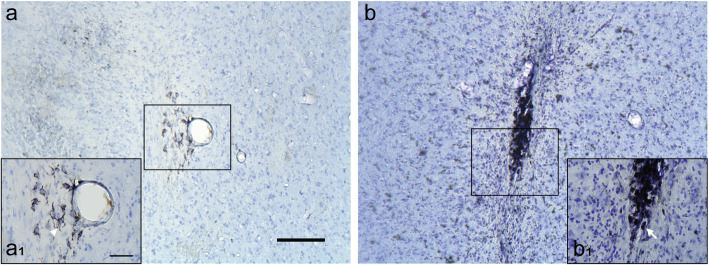
Brain tissue sections of the putamen immunostained against CD68 and counterstained with Nissl showing examples of resting/ramified microglia/macrophage (**a**, a_1_: monkey R2) and activated/amoeboid microglia/macrophage (**b**, b_1_: monkey R5). Insets (a_1_) and (b_1_) are higher magnification images of the regions demarcated by the squares. Scale bar: **a**, 200 μm; a_1_, 50 μm

Activated CD68-ir microglia/macrophages were defined as cells with dense rounded bodies (amoeboid conformation) and no or small thickened processes; resting microglia/macrophages were identified as small CD68-ir cells with thin ramifications [39, 40] (Fig. 1). Based on these criteria, the ipsi- and contralateral putamen of each brain tissue section was rated for CD68-ir microglia/macrophage activation from 0 to 4 using a semi-quantitative rating scale. The scoring system was as follows: 0 = none, no more than 4 individual, small amoeboid CD68 labeled microglia/macrophages throughout the entire putamen; 1 = weak, small amoeboid microglia/macrophages forming no more than 2 small, sparsely populated clusters, with no other microglia/macrophages scattered in the putamen; 2 = minimal, small amoeboid microglia forming no more than 5 small, more dense clusters, with some amoeboid microglia/macrophages scattered individually throughout a localized area of the putamen; 3 = moderate, slightly larger amoeboid microglia/macrophages, more numerous, may form small clusters, mostly spread throughout a localized area of the putamen; 4 = strong, large, dark, rounded microglia/macrophages forming dense clusters that accumulate into distinct, localized areas of dark CD68 immunoreactivity with some spread of smaller microglia outside of the clusters. The final score for the ipsi- and contralateral putamen of each subject was obtained by averaging the results of three tissue sections. An AI for CD68-ir was then calculated as the difference between the contra- and ipsilateral putamen scores, to provide a qualitative index for comparison with the [^18^F]FEPPA PET analysis.

### Statistics

Data collection and analysis were performed by investigators blind to the treatment. Statistical analyses were performed with R (v3.5.3). Results are presented as mean ± SEM. The *p* values for data sets were calculated using a two-tailed, paired sample Student’s *t* test. The relationship between [^18^F]FEPPA AI and CD68-ir AI was analyzed using Spearman’s correlations. A *p* < 0.05 was considered significant.

## Results

### Radiochemistry

The radiochemical yield of [^18^F]FEPPA was 25 ± 8% uncorrected for radioactive decay (ndc) from a total synthesis time of 78 ± 4 min. The retention time of [^18^F]FEPPA on semipreparative HPLC was 30.7 ± 1.0 min. The original method of FEPPA production reported yields of 50–60% ndc using a precursor mass of 5 mg [36]. After modifications to the reported synthesis with the addition of a cartridge-based SPE step, this same group reported yields of 30 ± 6% ndc [35]. With our new method, reducing the precursor mass by 60% produced similar yields to the previously published reports. From analytic HPLC, the retention time of product was 6.60 ± 0.01 min with a molar activity of 751.1 ± 162.8 MBq/nmol (at the end of synthesis) and radiochemical purity > 99%. The total impurity mass excluding peaks from the formulation vehicle was 2.51 ± 0.44 nmol determined through analytic HPLC. The radiotracer displayed no radiolysis for at least 4 h post-formulation and required no stabilizing agents.

### PET image analysis of [^18^F]FEPPA uptake

**Fig. 2:**
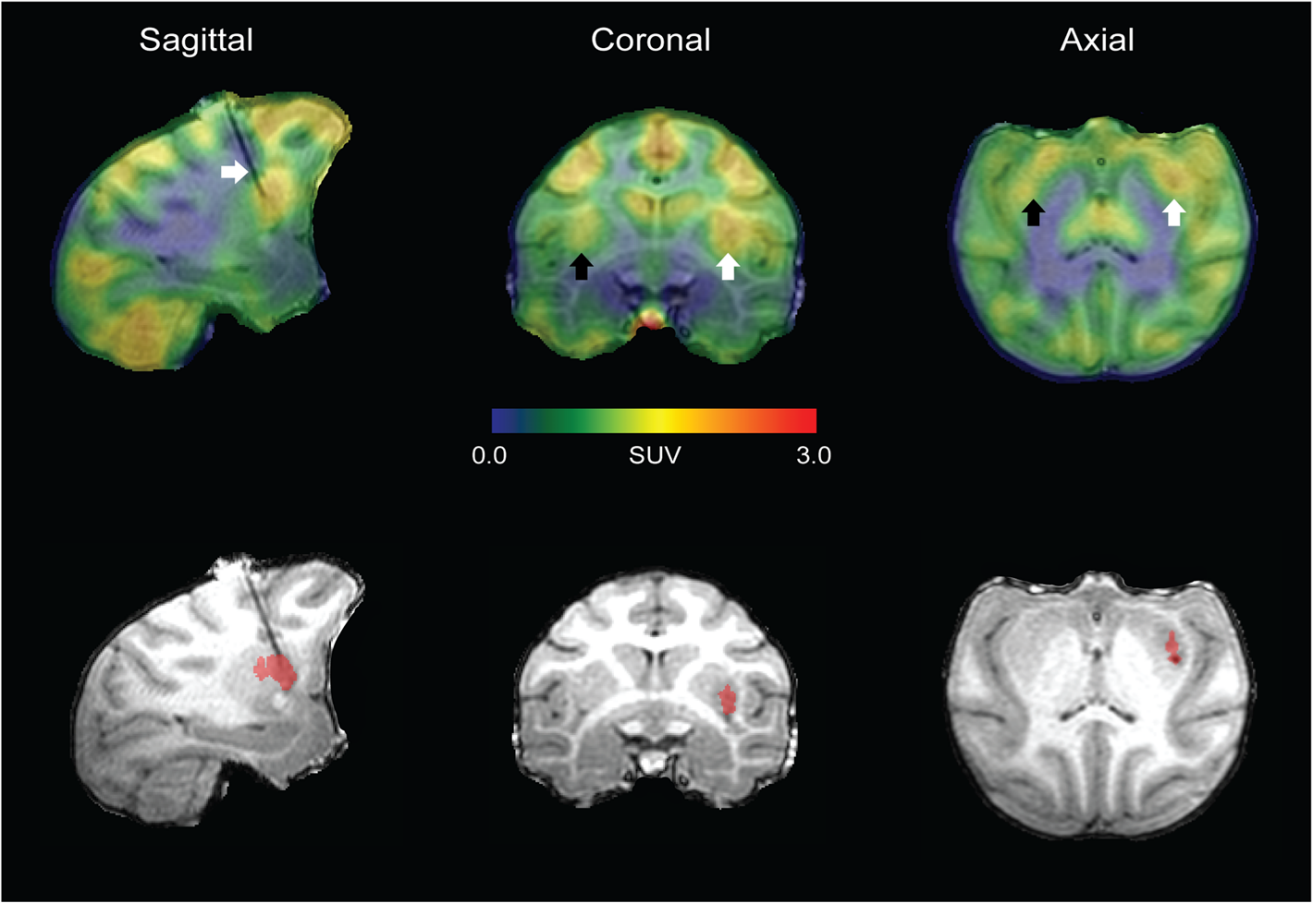
Representative [^18^F]FEPPA PET brain image of a rhesus macaque (R1) with allogeneic iPSC-mDA. Visual asymmetry in uptake is detected in the putamen of monkeys with allogeneic grafts. Images were overlayed on the MRI acquired during RT-IMRI delivery of the iPSC-mDA. Arrow in sagittal slice points to the cannula used for cell delivery. Arrows in coronal/axial slice point to the contralateral (left, black arrow) and ipsilateral (right, white arrow) putamen. The red overlay in the MRI is representative of the allogeneic iPSC-mDA graft and was utilized for visualization of [^18^F]FEPPA binding

**Fig. 3:**
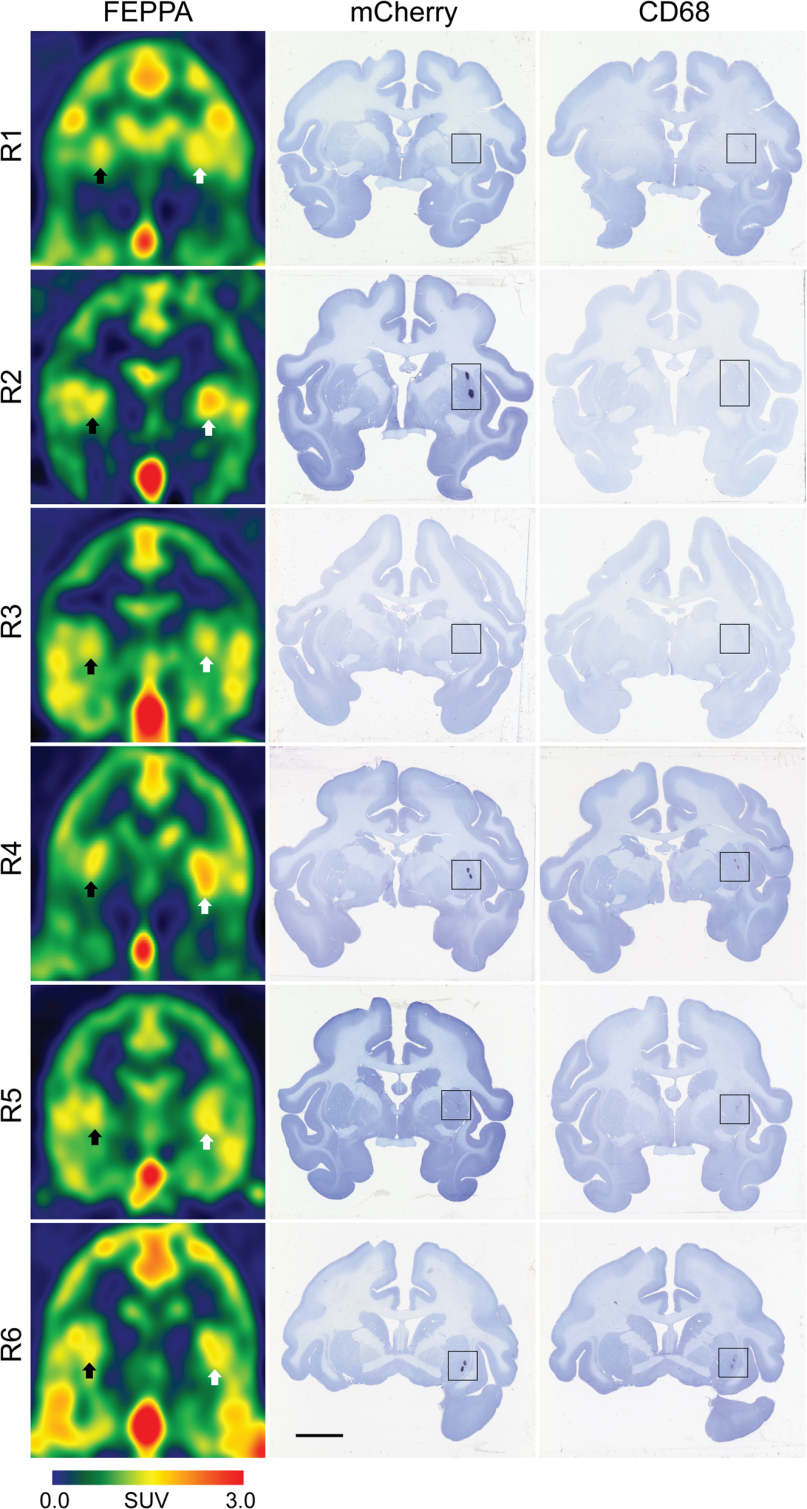
Coronal brain images of [^18^F]FEPPA PET and mCherry and CD68-immunostained tissue sections counterstained with Nissl for each of the six animals. Arrows point to the contralateral (left, black arrow) and ipsilateral (right, white arrow) putamen; black squares demarcate grafted immunoreactive area. Scale bar: 1 mm

**Fig. 4:**
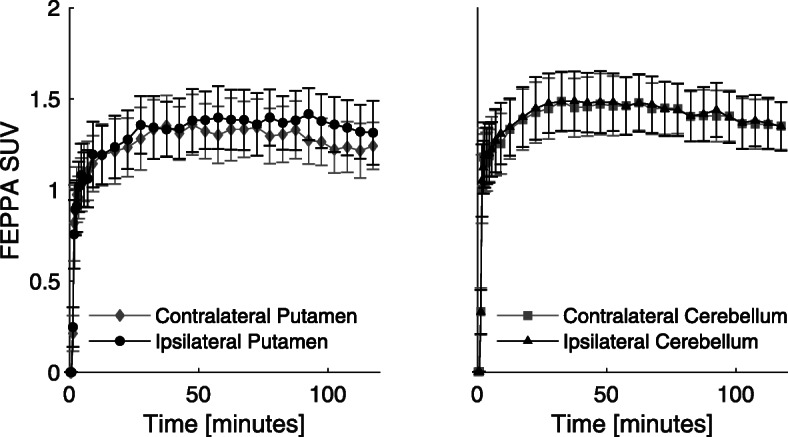
Mean [^18^F]FEPPA time-activity curves for monkeys (*n* = 6) receiving allogeneic iPSC-mDA. Error bars represent the standard error of the mean (SEM). Higher uptake is observed in the graft sites with allogeneic iPSC-mDA compared to the contralateral (control) putamen

From visual inspection of the data, putamen asymmetry of [^18^F]FEPPA uptake was observed in the rhesus with allogeneic iPSC-mDA (Fig. 2). PET images for all animals are presented in Fig. 3. Average time-activity curves show the differences in uptake of [^18^F]FEPPA in the ipsilateral putamen relative to the control, untreated putamen (Fig. 4) averaged over all of the subjects. The asymmetry index comparing the ipsilateral (right) and contralateral (left) regions generated for the putamen and cerebellum (control region anticipated to be unaffected by the grafts) of each animal are shown in Table 2. The monkeys revealed increased asymmetry of [^18^F]FEPPA uptake (*t* = 6.82; *p* = 0.001) in the putamen (− 0.085 ± 0.018), and no difference (− 0.007 ± 0.020) in uptake between the ipsi- and contralateral cerebellum, confirming that the putamen asymmetry observed was not a result of hemispheric image artifacts.

**Table 2:** Asymmetry index (AI) results for monkeys with allogeneic iPSC-mDA. Putamen asymmetry is detected due to the unilateral placement of allogeneic grafts, while no asymmetry is noticed in the cerebellum. AI between the putamen and cerebellum were compared using a paired *t* test

Monkey ID	Putamen AI	Cerebellum AI	*t* value	*p* value
R1	− 0.066	0.045		
R2	− 0.102	− 0.035		
R3	− 0.001	0.055		
R4	− 0.113	− 0.071		
R5	− 0.120	− 0.033		
R6	− 0.108	0.000		
Mean ± SEM	− 0.085 ± 0.018	− 0.007 ± 0.020	6.82	0.001

### Postmortem brain analysis

**Fig. 5:**
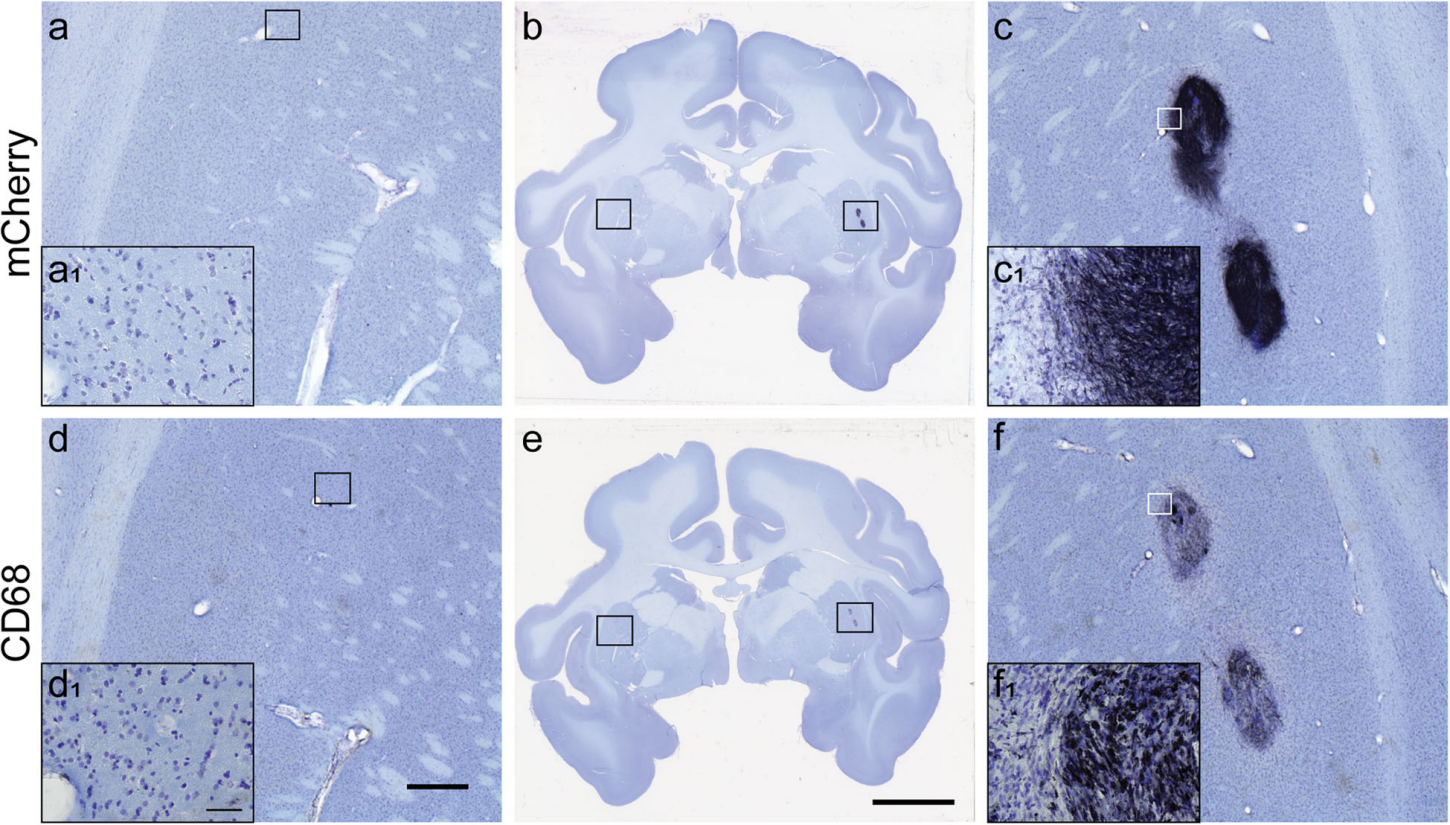
mCherry (**a**–**c**) and CD68 immunostained (**d**–**f**) brain tissue sections counterstained with Nissl from the hemiparkinsonian monkey R4 with unilateral allogeneic grafts in the postcommisural putamen. Images **a**, **c** and **d**, **f** correspond to higher magnification of the squares in coronal brain sections **b** and **e** respectively. Insets a_1_, c_1_, d_1_, and f_1_ are higher magnification of the square in panels **a**, **c**, **d**, and **f** respectively. Scale bars: **d**, 1 mm; **e**, 10 mm; d_1_, 50 μm

Evaluation of mCherry immunostaining by an independent investigator confirmed the unilateral presence of grafted cells in the RT-IMRI-targeted putaminal areas of all animals. mCherry-positive cells with neuronal-like morphology were found forming dense dark clusters, with minimal, short neurite extensions towards the rest of the host putamen (Fig. 5). mCherry and CD68-immunostained brain tissue sections for each animal are presented in Fig. 3.

**Fig. 6:**
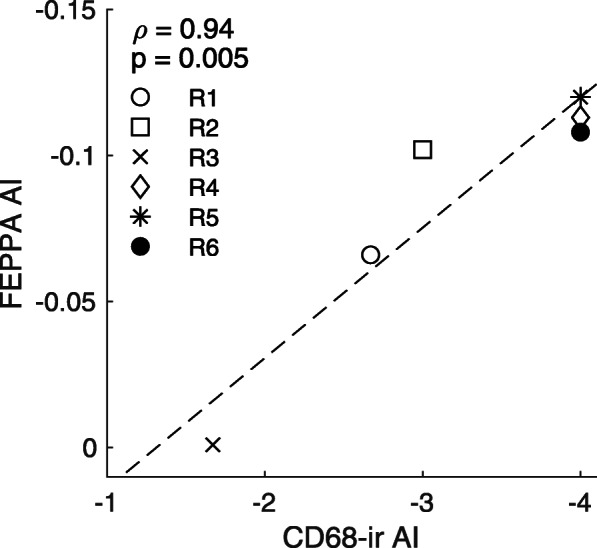
Spearman’s correlation between [^18^F]FEPPA AI and CD68 immunoreactivity (CD68-ir) AI for each of the six monkeys (Spearman’s *ρ* = 0.94, *p* = 0.005). AI < 0 indicates increased [^18^F]FEPPA/CD68-ir in the ipsilateral putamen

The pattern of CD68-ir presented differences within and between individuals. In all analyzed sections, the grafted ipsilateral putamen had CD68-positive cells that ranged from a couple of small clusters with few amoeboid microglia/macrophages (score 1) to strong CD68-ir clusters of large, dark, amoeboid microglia/macrophages and variable spread of smaller CD68-positive cells outside of the clusters (score 4; Fig. 5). In contrast, the untreated contralateral putamen had no CD68-ir as observed in Fig. 3, except one case (R1) in which 2 sections showed several weak CD68-ir cell clusters with spread out amoeboid microglia/macrophages (score 1). Blind quantification of CD68-ir with the rating scale identified significant differences in activated microglia/macrophage levels between the ipsilateral (3.33 ± 0.38 (mean ± SEM)) and contralateral (0.11 ± 0.11) putamen (*p* = 0.0004). Comparison between the CD68-ir AI and [^18^F]FEPPA AI showed a significant positive correlation (Spearman’s *ρ* = 0.94, *p* = 0.005; Fig. 6).

## Discussion

The goal of these imaging experiments was to assess whether TSPO PET with [^18^F]FEPPA can be used in translational studies as a sensitive in vivo measure of microglia/macrophage activation in the brain. As a study platform, we leveraged an ongoing project in our lab in which hemiparkinsonian rhesus macaques were treated with allogeneic iPSC-mDA and compared the in vivo [^18^F]FEPPA PET uptake to *postmortem* CD68 brain expression to further validate the PET imaging results.

Of significant translational interest is that [^18^F]FEPPA has been used to evaluate inflammatory cell recruitment in the brains of PD patients [41–43]. The PET studies successfully captured the mild neuroinflammatory changes induced by the disease, yet they did not identify a direct relationship between the amount of microglia/macrophage activation observed by [^18^F]FEPPA and PD progression. The results likely reflect stable activated microglia/macrophage recruitment over time, although nucleotide polymorphism in the TSPO gene (rs6971) found in humans of European heritage may complicate the interpretation of the results [25]. The monkeys in this study were rendered with stable hemiparkinsonism by intracarotid artery administration of the neurotoxin MPTP. Mild neuroinflammation is observed in the nigrostriatal system after a MPTP challenge [34] and persists for many years [44]. The ongoing MPTP-induced neuroinflammation may have contributed to the clear unilateral inflammatory response triggered by the allogeneic grafts and detected by [^18^F]FEPPA signal in all animals.

For this preclinical application of [^18^F]FEPPA PET, we also report strategies developed to improve and standardize radiotracer production. A method reported by Vignal et al. produced high yields of [^18^F]FEPPA (34 ± 2% ndc) using an ethanol-based separation and 5 mg of precursor without an SPE step [45]. A similar method using an ethanol-based separation but a lower reaction temperature (70 °C for 20 min) produced yields of 13 ± 8% ndc [46]. Compared to these methods and the initial synthesis by Wilson et al. [36], our radiochemical synthesis optimization allowed high yield production of [^18^F]FEPPA (25 ± 8% ndc) with a 60% reduction in precursor mass, thus decreasing the cost of production and reducing the analyte mass load on the HPLC column. With our method, the use of an additional cartridge-based SPE step prior to semipreparative HPLC ensured no free [^18^F]-fluoride or residual kryptofix222 appeared in the final product, while further reducing the HPLC analyte mass load. The use of acetonitrile in the semipreparative HPLC mobile phase resulted in a clearly defined separation of product from impurities. Although the retention time of [^18^F]FEPPA increased with this semipreparative HPLC separation, the final product solution contained very low impurity mass, providing an alternate method of manufacturing that is suitable for nonhuman primates and future human studies.

The lack of a more quantitative index of TSPO expression using an arterial derived input function (e.g., binding potential or volume of distribution) is a limitation of this work and prevented the ability to make absolute (i.e., unnormalized) comparisons of PET- and CD68-measured outcomes. The [^18^F]FEPPA data were analyzed using standardized uptake values (SUVs) of static images averaged across the 90–120 min image frames. The use of an asymmetry index was chosen as the metric to best interpret the data since each monkey contained a control region without the presence of iPSC-mDA grafts to compare against. The SUV (=PET concentration/injected dose/body weight) is a simplified and commonly used proxy for receptor specific binding metrics (e.g., DVR, BP_ND_, BP_F_), obviating the need for an arterial derived input function. In practice, it is more frequently implemented as a ratio (SUVR) of target and reference region concentrations after a sufficiently long period of uptake to minimize variations due to blood flow. The asymmetry index used in this application as the outcome measure represents a ratio of SUVRs (i.e., (L-R)/((L + R)/2)) in bilateral (left, right) brain regions. As such, the need for a reference region is circumvented because it appears in both the numerator and denominator. Thus, many of the biases accompanying the SUV index are minimized using the asymmetry index, assuming the bilateral regions have highly similar radiotracer delivery and binding under typical conditions (i.e., in the absence of neuronal grafts). To test this assumption, the asymmetry index was also determined in an alternate brain region (bilateral cerebellar cortex) that was unaffected by surgery.

Increased ipsilateral [^18^F]FEPPA uptake, indicated by AI < 0, correlated with higher CD68 AI ratings, suggesting that increased TSPO expression measured by PET corresponds to increased recruitment of activated microglia/macrophages due to the allogeneic nature of the grafts. Numerous mCherry-positive rhesus iPSC-mDAs successfully survived 24 months after brain surgery without immunosuppression. The grafted cells were localized in the targeted putamen areas, forming tight clusters with short neurite extensions (a full description of the grafts and graft survival is out of the scope of this paper and will be published elsewhere). These results differ from our previously reported poor survival of human-derived embryonic SC-mDAs in cyclosporine-immunosuppressed PD monkeys [47]. In that study, 3 months after brain surgery, the xenograft recipients presented signs of neuroimmune rejection observed as copious amounts of CD68-ir, with activated microglia/macrophages in areas of necrosis and spread across the basal ganglia.

In the current study, the CD68 immunostaining was blindly analyzed using a semi-quantitative rating scale for activated microglia/macrophage assessment. We chose this method because the inflammatory reaction was minimal and attempts to obtain optical density standardized measures of CD68 expression in the putamen were not sensitive enough to detect the subtle differences in cellular morphology and number. Since the CD68 is an accepted marker of activated microglia/macrophages [17] and the ratings considered the accumulation of microglia/macrophages with activated morphology, these results further suggest that [^18^F]FEPPA signal in these regions correspond to the presence of activated microglia/macrophages. The neuroinflammation associated with the allogeneic grafts was subtle and mostly localized, yet [^18^F]FEPPA PET demonstrated sufficient sensitivity to detect it.

We cannot rule out that astrocytic response may have contributed to the [^18^F]FEPPA signal. TSPO overexpression has been described in reactive astrocytes [48, 49] and GFAP immunoreactive cells were observed in the grafted areas. Follow-up studies exploring the relationship between TSPO, [^18^F]FEPPA, and GFAP+ cells are warranted.

The correlation between TSPO PET and CD68-ir is not unique to this study, as English et al. showed that increased uptake of [^11^C]PBR28 corresponded with a higher CD68 rating in a rat model of aortic aneurysm [50]. Hannestad et al. reported that baboons treated systemically with *E. coli* lipopolysaccharide presented an increase in [^11^C]PBR28 uptake that positively correlated with serum cytokines IL-1β and IL-6 levels. This finding was associated with increased TSPO-ir that was mainly present in activated CD68-positive microglia/macrophages [51]. These studies provide further evidence that the increase in [^18^F]FEPPA uptake observed in the putamen with allogeneic grafts is a result of increased microglia/macrophage activation.

With iPSC-derived lines showing strong promise in translational studies aiming to replace cells lost to neurodegeneration, the need for in vivo methods to assess the host’s immune response against grafted cells has emerged. Kikuchi et al. reported that human iPSC-derived dopaminergic progenitors survived and induced functional recovery in FK506-immunosuppressed monkeys with MPTP-induced Parkinsonism [52]. When evaluating the immune response to these cells via [^11^C]PK11195 PET, the authors described either no inflammation or mild inflammation in grafted areas [52]. Additionally, use of S-[^11^C]KTP-Me PET to monitor cyclooxygenase-1 (COX-1) revealed no change in uptake, suggesting absence of microglial response. The *postmortem* analysis found none to minimal CD45 hematopoietic T cell immunoreactivity, but abundant MHC II expressing immune cells, indicating the lack of sensitivity of [^11^C]PK11195 and S-[^11^C]KTP-Me for detecting the recruitment of microglia and its metabolic activity [52]. It has been reported that [^11^C]PK11195 has low brain uptake in both humans and nonhuman primates [53, 54], low sensitivity to detect TSPO, and high lipophilicity resulting in nonspecific binding to lipids in the brain which can interfere with PET quantification [55]. Compared to second generation TSPO radioligands such as [^11^C]PBR28, [^11^C]PK11195 specific binding in the brain is 80-fold lower in rhesus macaques [56]. Our study has demonstrated that [^18^F]FEPPA has the sensitivity to detect CD68-positive activated microglia/macrophages following grafting of allogeneic iPSC-mDA while showing high uptake in the brain, holding promise for clinical applications in monitoring immune response.

## Conclusion

PET imaging with [^18^F]FEPPA is a sensitive method for evaluating in vivo CD68-positive microglial/macrophage activation in the brain as demonstrated by the correlation between in vivo uptake of the radioligand and *postmortem* CD68 immunoreactivity ratings. These findings in rhesus macaques with allogeneic grafts of iPSC-mDA suggest that [^18^F]FEPPA holds promise for in vivo monitoring of immune response in clinical applications.

## Data Availability

The datasets generated during and/or analyzed during the current study are available from the corresponding author on reasonable request.
